# Disentangling the Therapist Effect: Clustering Therapists by Using Different Treatment Outcomes

**DOI:** 10.1007/s10488-024-01365-3

**Published:** 2024-03-21

**Authors:** Pauline Janse, Naline Geurtzen, Agathe Scappini, Giel Hutschemaekers

**Affiliations:** 1 Pro Persona Research, Wolfheze, The Netherlands; 2 Behavioural Science Institute, Radboud University, Nijmegen, The Netherlands

**Keywords:** Therapist effects, Cluster analysis, Treatment outcomes, Psychotherapy outcomes, Learning activities

## Abstract

Previous studies have shown that therapists’ performance varies, known as therapist effects, and have indicated that therapists who excel in one treatment outcome may not necessarily be effective in other outcomes. This observational naturalistic study aimed to enhance our understanding of therapist effects and the assessment of therapists’ performance in different areas. The study included 68 therapists and 5,582 clients from a large mental health facility. Information about their learning activities was available for a subsample of 49 therapists. Separate multilevel analyses were conducted for treatment outcomes, including case mix-corrected OQ-45 change scores, dropout rates, referrals to other facilities, treatment duration, and client satisfaction ratings. A hierarchical cluster analysis was performed to identify groups of therapists based on their performance across various treatment outcomes. Additionally, differences in therapist characteristics among the clusters identified were examined. Therapist effects varied across different outcomes, ranging from small (2.6% for OQ-45 change) to moderate (6.5% for number of sessions). The cluster analysis revealed four distinct clusters of therapists with specific profiles. They had performance differences in certain areas but not in others. This exploratory study supports the notion that therapists exhibit diverse profiles regarding treatment outcomes. These findings are significant for future investigations of therapist effects that aim to identify the characteristics of effective therapists and in the context of personalizing treatment for clients.

**Supplementary Information:**

The online version contains supplementary material available at 10.1007/s10488-024-01365-3.

## Introduction

Therapist effects (TEs) refer to the systematic differences among therapists in their effectiveness (Wampold et al., 2017). Investigating TEs can provide insights into therapists’ characteristics that contribute to their success, such as interpersonal skills (Anderson et al., 2016), responsiveness (Stiles et al., 1998; Watson & Wiseman, 2021), and deliberate practice (Chow et al., 2015). The ultimate objective is to understand the underlying mechanisms of psychotherapy and develop tools to enhance therapists’ skills and, consequently, the effectiveness of their therapies.

The current evidence, however, remains inconclusive because numerous variables obscure a precise understanding of TEs. Studies vary widely in the reported magnitude of TEs, which is expressed as the percentage of variance attributed to therapists. For instance, in a review by Johns et al. (2019), TEs ranged from 0.2 to 30%. Apart from specific therapist characteristics, Johns et al.‘s (2019) study suggests that differences in TEs are also influenced by the specific therapies employed (treatment effects), the clients treated (client effects), the working environment (setting effects), and interactions among these variables. For example, Firth et al. (2020) demonstrated significant variations among settings, with the largest TEs observed in primary and secondary care and the smallest in University-based clinics, Voluntary (e.g., charitable organizations), and Workplace (e.g., treatment offered by an employing organization). Without understanding the exact contributions of these confounding factors, interpretation of the discrepancies in reported TEs is challenging, and replicating outcomes of studies investigating therapist characteristics that can improve therapists’ performance is very difficult. For instance, Janse et al. (2023) were unable to replicate Chow’s results on deliberate practice, which may be attributed to differences among therapies, clients, settings, or a combination of these factors. Achieving a clear estimation of TEs requires all these variables to be controlled; however, doing so would be quite complex without having detailed information about the exact nature of these variables.

Another major challenge in investigating TEs is how the concept should be operationalized. Typically, TEs are defined as the percentage of variance in clients’ symptom reduction that can be explained by therapists’ factors. This operationalization is, however, too narrow. Other outcome variables, such as the proportion of treatment dropouts (i.e., premature unilateral terminations), are also clinically relevant. A meta-analysis conducted by Fernandez et al. (2015) revealed that more than one-fourth of the clients receiving cognitive-behavioral therapy dropped out of treatment, indicating that a considerable proportion of clients do not receive the needed assistance. Therapists can have a significant impact on dropout rates, as evidenced by research reporting that TEs in dropout rates range from 5.7% (Zimmermann et al., 2017) to 12.6% (Saxon et al., 2017). Other relevant outcome measures include treatment duration and the number of sessions required by clients. While shorter treatment is not an objective in itself, the context of extensive waiting lists for mental health care (e.g., BMJ, 2022) and the potential adverse effects of prolonged waiting times on treatment outcomes (Van Dijk et al., 2023) underscores the necessity of regarding treatment duration as an important variable. Lutz et al. (2015) demonstrated that therapist variance is more significant for treatment length than symptom reduction (8.89% versus 5.88%, respectively). However, therapists who see clients longer are not necessarily more effective. On the other hand, certain therapists might be more inclined to refer clients elsewhere if an initial intervention proves ineffective, though, to our knowledge, this possibility has not been investigated.

The challenge with all the potential outcome measures is to identify which therapist characteristics predict different effects. As demonstrated by Lutz et al. (2015), therapists who excel with one outcome measure may not demonstrate the same proficiency with others. Further analysis of their data revealed no correlations between therapists’ effectiveness, treatment length, or dropout rate (Barkham et al., 2017, p.46), highlighting the need for additional research. These results suggest that, rather than evaluating therapists’ performance based on a single outcome measure, systematic interactions between outcomes and therapists’ characteristics should be investigated. This approach could enable us to identify different therapist groups with specific profiles who are effective in one area but not others. To our knowledge, no study has investigated this possibility.

This naturalistic observational study aimed to examine therapists’ performance in different areas within a homogeneous therapeutic context. First, we explored TEs, considering not only symptom reduction but also dropout and referral rates, client satisfaction, and treatment duration. Second, our investigation focused on discerning whether different groups of therapists could be identified based on their performance in these areas.

## Method

### Setting

This study utilized data from clients in a large mental health facility in the Netherlands who received treatment between January 2014 and November 2018. The treatments were part of what is known as *basic mental health care* in the Netherlands. These brief therapies were readily available to clients referred by their general practitioner, covered by health insurance, and without lengthy waiting times. The treatment consisted of brief integrative therapy (Rijnders & Heene, 2015), which combined cognitive behavioral therapy, acceptance and commitment therapy, and solution-focused interventions (Hutschemaekers et al., 2019).

### Therapists

The original database included 109 therapists who treated 5,891 clients. Following Janse et al.‘s (2023) approach, we selected therapists who had treated a minimum of 10 clients to insure reliable judgments of therapists’ outcomes; this resulted in a final sample of 68 therapists. The therapists had a mean caseload of 82.1 clients (range 11–296, median = 57.5). Of the therapists, 15 were male (22.1%) and 53 were female (77.9%). Among the 68 therapists, 50 (73.5%) were certified health psychologists, 9 (13.2%) were in training to become certified health care psychologists, and 9 (13.2%) were master’s level psychologists. Becoming a certified health care psychologist is a formal post-masters’ professional certification and a requirement in the Dutch health care system to be able to work independently as a therapist. The training entails an intensive, full-time, two-year theoretical and practical education, during which therapists receive supervision in developing their therapeutic skills. On average, the therapists had 9.78 years of experience, and they conducted 19.57 sessions per week.

### Clients

A total of 5,582 adult clients were included in the analyses. They had various psychological disorders, with mood and anxiety disorders being the most common examples. Clients who at intake exhibited acute suicidality, psychosis, or severe substance abuse were excluded from treatment and referred to specialized services. Table 1 provides an overview of the clients’ characteristics.

**Table 1:** Characteristics of the clients (*N* = 5,582)

Characteristics	
Age *M* (*SD*; range in years)	38.9 (14.3; 18–94)
Gender	
Female (*N* = 3318), %	59.4%
Male (*N* = 2241), %	40.1%
Missing (*N* = 23), %	0.4%
Marital state	
Unmarried (*N* = 3162), %	56.6%
Married (*N* = 1730), %	31.0%
Divorced (*N* = 563), %	10.1%
Widowed (*N* = 124), %	2.2%
Missing (*N* = 3), %	0.1%
Employment	
Unemployed (*N* = 2269), %	40.6%
Employed (*N* = 3310), %	59.3%
Missing (*N* = 3), %	0.1%
Education	
Primary school (*N* = 432)	7.8%
Secondary or vocational (*N* = 3985)	71.4%
University (*N* = 1155), %	20.7%
Missing (*N* = 10), %	0.2%
Average duration of symptoms pretreatment	
Longer than a year (*N* = 3170), %	56.9%
Mood disorders (*N* = 2156), %,	38.6%
Anxiety disorders (*N* = 1693), %,	30.3%
Trauma and stress-related disorders, (*N* = 700), %,	12.5%
Somatoform disorders (*N* = 392), %,	7.0%
Developmental disorders (*N* = 277), %	5.0%
Obsessive-Compulsive and Related Disorders (*N* = 78), %	1.4%
Personality disorders (*N* = 49), %,	0.9%
Other (*N* = 147), %	2.6%
Missing (*N* = 90), %	1.6%

*Notes* M = mean; SD = standard deviation

### Measures

#### Client Measures

The Outcome Questionnaire-45 (OQ-45, Lambert et al., 2004) is a self-report measure comprising 45 items scored on a five-point Likert scale ranging from *never* (0) to *almost always* (4). It was specifically designed to capture changes occurring during psychotherapy. Total scores can range from 0 to 180, with higher scores indicating more complaints or greater levels of distress. The items are grouped into three subscales: (a) Symptom Distress, (b) Interpersonal Relationships, and (c) Social Role Performance. The psychometric properties of the Dutch version of the OQ-45 are satisfactory and like those of the original instrument and include good internal consistency (for the total score, Cronbach’s *α* = 0.93). The cut-off between normal and clinical samples has been established as 56, and the reliable change index for a clinical population has been calculated as 18 (Timman et al., 2017).

At the end of the clients’ treatment, therapists documented in each client’s electronic file the reason for terminating. Clients who unilaterally ended treatment prematurely or did not keep appointments and stopped responding to requests for contact were designated as *dropouts*. Clients who during treatment were referred elsewhere for treatment were also documented in the client’s electronic file.

Clients’ satisfaction with their treatment was assessed using a single item from the Consumer Quality Index (CQI; Van Wijngaarden et al., 2007). Using this item, clients rate their treatment on a scale from *1* to *10*, with *1* indicating *very poor* and *10* indicating *excellent* treatment. Developed by the Dutch Institute for Research on Healthcare (NIVEL), the CQI is used to evaluate the quality of outpatient mental health treatments and is used throughout the Netherlands.

The duration of treatment was quantified based on the total number of sessions that the client attended, which was extracted from the client’s electronic file. Therapists’ mean number of sessions and duration of treatment were calculated in two ways: based on the total sample of clients and on clients who completed the therapy because most clients who dropped out did so early in the treatment.

Clients’ perceived overall health status was evaluated using the EQ-VAS, which is part of the EQ-5D (Feng et al., 2021). The EQ-VAS is a Visual Analogue Scale, which allows clients to rate their self-perceived health on a scale ranging from *0* (*the worst health you can imagine*) to *100* (*the best health you can imagine*).

In addition to completing these questionnaires, as part of the standard care, clients were asked to indicate their demographic characteristics, including age, gender, duration of symptoms, employment status, educational level, and marital status.

#### Therapists’ Characteristics

The Retrospective Analysis of Psychotherapists’ Involvement in Deliberate Practice (RAPID Practice; Chow & Miller, 2016) is a survey that gathers information about therapists’ work practices, their views on professional development, and the time they spend on activities aimed at improving their therapeutic skills. It includes 25 items that assess the amount of time therapists believe they spend on enhancing their therapeutic skills within three domains: solitary activities, non-solitary activities, and non-therapeutic activities. Therapists indicate the amount of time spent in the latest typical working month and the relevance of each activity for improving their clinical skills. Additionally, they indicate the average number of hours they are dedicating per week in the current year to activities associated with honing their therapeutic skills.

### Procedure

Clients were referred to the treatment facility by their general practitioner. Each client was then allocated to a therapist according to therapists’ availability. This process often led to a rather arbitrary assignment of clients to therapists. When the therapist assigned was either a masters-level psychologist or was in training to become a certified psychologist, a psychologist who was already certified provided supervision.

As part of Routine Outcome Measurement (ROM), clients digitally completed both the OQ-45 and the EQ-VAS at the beginning and end of treatment. Also, at the end of the treatment clients were asked to complete the Consumer Quality Index.

### Statistical Analyses

Treatment outcome was determined by OQ-45 change scores, whether clients had dropped out of treatment, whether they had been referred elsewhere, the total number of sessions attended, and clients’ rating of their satisfaction at the completion of the treatment. Both TEs and mean scores on treatment outcome were calculated, and the latter were entered as the dependent variables in a cluster analysis.

Two other kinds of analyses were performed. First, multilevel analyses were used to determine variability among therapists (i.e., TEs) in their clients’ treatment outcomes. Intraclass correlation (ICC) was used as the measure of TEs. For each outcome variable, separate two-level analyses were performed. To calculate ICCs for the analysis in which dropouts was the binary outcome, 3.29 was used as the level-1 error variance (O’Connell et al., 2008). In the other two-level analyses, a random intercept model was used with the Maximum Likelihood method and an unstructured variance-covariance matrix for estimating random effects. The Akaike Information Criterion (AIC) and Bayesian Information Criterion (BIC) in the models were inspected to see whether random slopes in the covariates should be included in the model (with smaller numbers indicating a better model). All the continuous predictors were centered.

Next, aggregated scores for therapists were calculated. Similar to the approach in Brown et al.‘s (2005) study on identifying highly effective therapists and consistent with Janse et al. (2023), therapist effectiveness in symptom reduction was assessed by the therapists’ average residualized change score on the OQ-45 rather than solely relying on the average raw score difference between intake and post-treatment OQ-45. This method was employed because of the significant correlation between OQ-45 pre-treatment scores and OQ-45 change scores (which was not significant with the other outcomes) and to prevent the influence of differences in the types of patients seen by different therapists (i.e., case mix) from confounding the therapists’ average outcomes. The residuals, referred to as case mix corrected post-treatment OQ-45 scores hereafter, were computed using a two-level analysis with the OQ-45 pre-treatment score, the client’s age, symptom duration, and employment status as predictors. Subsequently, the aggregated scores included therapists’ mean case-mix-corrected OQ-45 change scores, the mean effect size (*d*; Morris & DeShon, 2002) for each therapist, the percentage of each therapist’s caseload that discontinued treatment, the percentage of each therapist’s caseload referred elsewhere, the mean number of sessions (based solely on clients who completed treatment to mitigate dropout bias), and the mean client satisfaction rating. Spearman’s *rho* was used to compare these outcome variables.

Third, as a separate step in analyzing the data, hierarchical cluster analyses (e.g., Yim & Ramdeen, 2015) were performed to determine whether there were distinct therapist groups based on therapists’ mean case-mix-corrected OQ-45 change scores, percentage of dropouts and referrals, number of sessions, and client satisfaction scores. To identify possible outliers, a hierarchical cluster analysis using a single linkage (nearest neighbor) method was performed, and then identified outliers were removed. In a subsequent hierarchical cluster analysis, Ward’s method with squared Euclidean distances (Murtagh & Legendre, 2014) was used. The optimal number of clusters was determined based on a visual inspection of the dendrogram from the cluster analysis; subsequently, several different methods of determining the number of clusters (Charrad et al., 2014) were compared.

Finally, differences among the various outcomes were evaluated, as were the available client variables among the clusters using Kruskal-Wallis tests. These tests were chosen as an alternative to ANOVA because they are suitable for cases with small sample sizes and ordinal dependent variables, as we observed in the subsample analyses. When the Kruskal-Wallis omnibus test indicated significant differences among the clusters, subsequent post hoc pairwise comparisons were conducted using an adjusted *p*-value based on Bonferroni corrections.

The analyses were performed using SPSS version 28 and R 3.4.0 using the R packages *tidyverse* for data manipulation, *cluster* for the clustering algorithms, *factoextra* for clustering visualization and for identifying the ideal number of clusters for a particular clustering method. “NbClust” (Charrad et al., 2014) was used to calculate approximately 30 methods simultaneously in order to determine the optimal number of clusters, and the *fpc* package was used for bootstrap resampling to evaluate how stable each cluster was.

## Results

### Descriptive Statistics

Clients’ pre-treatment OQ-45 total scores were consistent with those of other clinical samples (e.g., de Jong et al., 2007). They indicated elevated levels of symptom distress and interpersonal difficulties and diminished quality of life. By the end of treatment, the OQ-45 scores showed, on average, a reliable improvement (i.e., of more than 18 points; Timman et al., 2017) with treatment and yielding a large effect size. Clients who had dropped out of therapy or were referred elsewhere during therapy achieved less change (effect size *d* = 0.56 and *d* = 0.02). On average, clients received 8.21 therapy sessions (*SD* = 3.78). Among clients who dropped out of therapy, the average number of sessions was 5.30 (*SD* = 3.08), while those referred elsewhere for treatment during therapy received an average of 8.23 sessions (*SD* = 4.55). Table 2 shows the descriptive statistics for the measures of treatment outcome.

**Table 2:** Clients’ mean scores at pre- and post-treatment

	M (SD; range)
OQ-45 pre-treatment (*N* = 4678)	80.47 (22.33; 8-164)
OQ-45 post-treatment (*N* = 2835)	60.45 (25.92; 2-162)
Effect size *d* (*N* = 2469)	0.80 (0.91; -3.61-4.44)
Client satisfaction (*N* = 3170)	8.19 (1.54; 0–10)
EQ-VAS pre-treatment (*N* = 3699)	60.11 (20.30; 1-100)
EQ-VAS post-treatment (*N* = 3378)	71.97 (18.75; 1-100)
Drop-out (*N* = 547), %	17.1%
Referrals (*N* = 400), %	11.3%

### Therapist Effects

#### OQ-45 Change Scores

The calculation of Therapist Effects (TE) and Intraclass Correlation Coefficients (ICCs) in a multilevel or mixed effects linear regression model was carried out without using predictor variables. The TE for OQ-45 change scores was 2.6%. When significant covariates were included, the TE was reduced to 2.1%. Including a random slope (OQ-45 pre-treatment scores) indicated a more favorable model fit, as evidenced by lower AIC and BIC values. For detailed output and estimates of the OQ-45 change score analysis models, see the supplementary materials (Tables 1a - 1c).

#### Treatment Duration

In the unconditional model without predictors, TEs for the number of sessions was 7.2%. However, considering the relationship between dropouts and the number of sessions, TEs were also examined after clients who had dropped out of treatment were excluded. This resulted in a TE of 6.5% (5.5% when adjusted for OQ-45 pre-treatment scores). The addition of random slopes did not yield significant improvement in the model.

#### Clients’ Treatment Satisfaction Ratings

In the unconditional model, TEs for client satisfaction was 3.8%, but this was reduced to 3.2% when the significant covariates were included (see Tables  3 and 3b in the supplementary materials). Again, the model without random slopes had the best fit.

**Table 3:** Therapists’ mean treatment outcomes

	M (SD)	Minimum	Maximum
Effect size d	0.78 (0.25	0.32	1.05
OQ-45 change scores*	0.37 (5.44)	-14.31	14.12
Number of sessions	8.87 (1.10)	6.05	11.52
Client satisfaction score	8.19 (0.42)	6.82	9.05
Percentage of dropout in caseload	15.24 (7.86)	0.00	40.00
Percentage of referrals in caseload	11.51 (5.85)	1.85	28.07

* residualized change scores

#### Dropouts

The unconditional multilevel model with a binary outcome yielded TEs of 4.7%. Including covariates in the model yielded TEs of 5.2% (see Table 5 in the supplementary materials).

#### Referrals

The therapists in the unconditional multilevel model with a binary outcome yielded a TE of 5.1%. When the covariates were included in the model, the TE was also 5.1% (see Table 5a and 5b in the supplementary materials).

The therapists’ outcomes are further described in Table 3. The *rho* correlations between mean therapist’s treatment outcomes ranged between 0.01 and − 0.32. The missing OQ-45 change scores of clients within the therapists’ caseload ranged from 15.1 to 61.4% (*M* = 37.9%, *SD* = 9.6), and this was significantly correlated with the percentage of clients in the therapists’ caseload who were referred elsewhere (*r*_*s*_ = 0.26, *p* = .033).

### Cluster Analysis

**Fig. 1:**
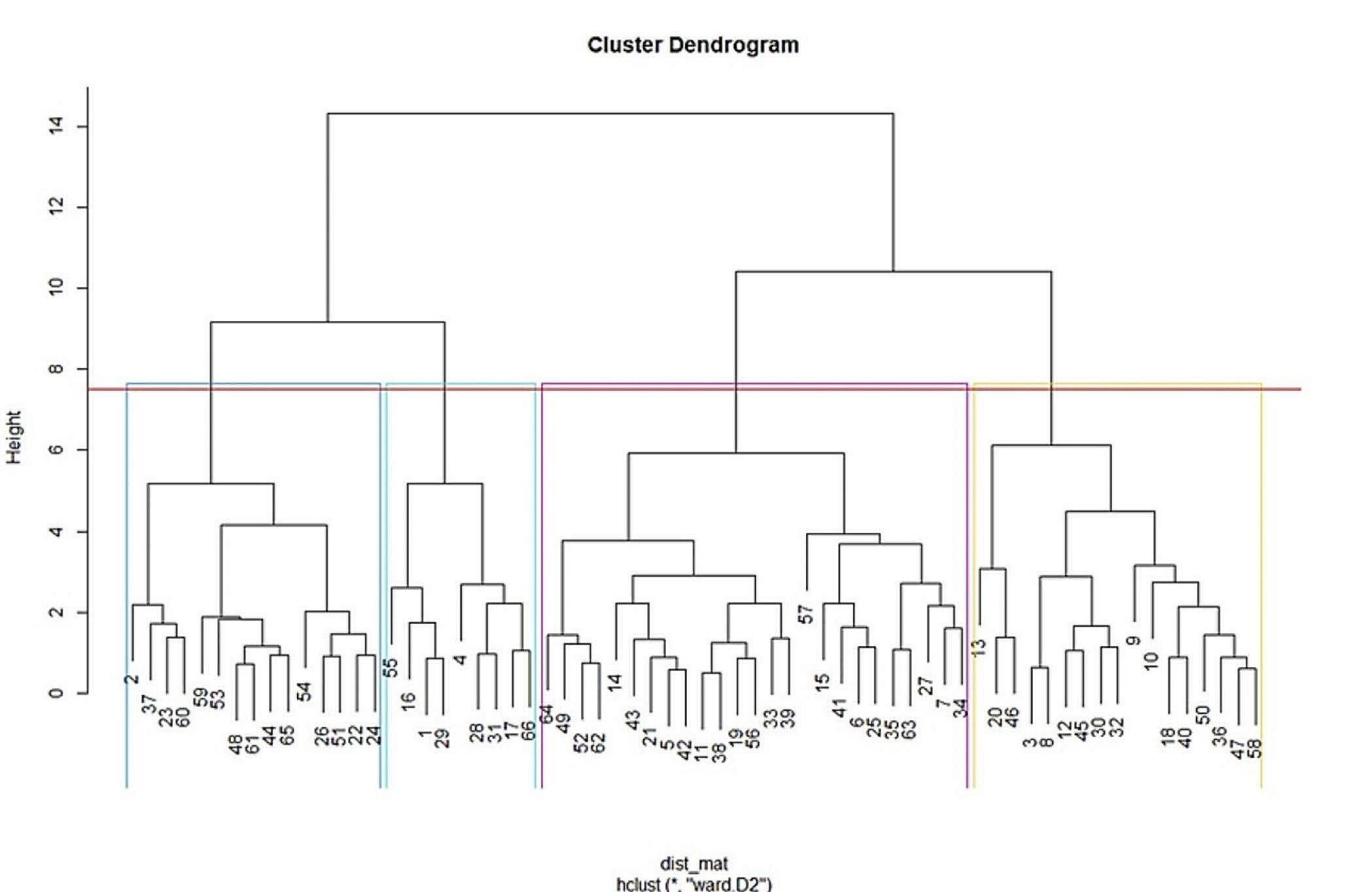
Dendrogram of the hierarchical cluster analysis based on therapists’ mean case-mix-corrected post-treatment OQ-45 scores, dropouts, referrals, number of sessions, and client satisfaction. *Note.* Each number on the horizontal axis corresponds to a therapist. The vertical axis specifically indicates the level of similarity at which the clusters were combined. Higher points on the axis indicate greater dissimilarity or distance between clusters; lower points indicate greater similarity

A hierarchical cluster analysis was performed that utilized aggregated therapist variables, including case-mix-corrected OQ-45 change scores, percentage of dropouts, percentage of referrals, treatment duration, and client satisfaction. The analysis resulted in a dendrogram, in which a four-cluster solution seemed most robust (see Fig. 1.). According to the *majority rule* in the results of the *NbClust* package, which employs 30 different methods to determine the optimal number of clusters, the optimal number of clusters was found to be four (see Figs. 1 and 2 in the supplemental materials for the results of the Elbow and Silhouette method). Assessment of cluster stability by resampling showed that average Jaccard similarities was greater than 0.85, which indicated highly stable clusters (Hennig, 2007).

Kruskal-Wallis tests with the four clusters as the grouping variable showed significant differences among the groups in their case-mix-corrected OQ-45 change scores [H(3) = 36.15, *p* < .001], Cohen’s *d* [H(3) = 27.09, *p* < .001], percentage of dropouts [H(3) = 40.80, *p* < .001], percentage of referrals, [H(3) = 24.16, *p* < .001], number of sessions [H(3) = 12.32, *p* = .006], and client satisfaction scores [H(3) = 10.04, *p* = .018]. As expected, there were no differences among the clusters on any of the other relevant client variables because several of the client factors had been controlled. Specifically, there were no notable distinctions among the clusters on EQ-VAS pre-treatment scores [H(3) = 2.86, *p* = .414], OQ-45 pre-treatment scores [H(3) = 0.61, *p* = .895], percentage of clients in paid employment [H(3) = 3.06, *p* = .383], nor duration of clients’ symptoms [H(3) = 0.44, *p* = .932] or in the age of the clients [H (3) = 4.09, *p* = .252]. Table 4 shows the number of therapists and the mean scores and standard deviations for the significant therapist variables in each of the four clusters.

**Table 4:** Descriptive statistics for the significant therapist variables in the four clusters

			OQ-45 change		Effect size d		Drop out %		Number of sessions		Client satisfaction		Referrals	
		*N*	*M*	*SD*	*M*	*SD*	*M*	*SD*	*M*	*SD*	*M*	*SD*	*M*	*SD*
Cluster	1	9	3.67	3.02	0.67	0.17	14.28	5.49	9.46	1.43	8.27	0.27	22.54	3.34
	2	15	-2.26	3.61	0.90	0.17	6.80	3.81	9.56	0.74	8.31	0.35	10.29	4.97
	3	17	5.95	3.77	0.55	0.19	13.38	4.63	8.59	0.96	7.89	0.52	8.79	3.08
	4	26	-2.80	4.36	0.91	0.22	21.67	6.57	8.44	1.01	8.29	0.35	10.17	3.90
Total		67	0.41	5.43	0.81	0.25	11.99	7.86	8.88	1.10	8.22	0.42	11.51	5.85

*Note.* lower case mix-corrected post-treatment OQ-45 scores indicate better outcomes

### Description of the Cluster Solutions

Post-hoc pairwise comparisons between the clusters were performed, and the results are presented in Tables  6, 7, 8, 9, 10 and 11 in the online supplemental materials. The four-cluster solution showed that Cluster 2 (*n* = 15) and Cluster 4 (*n* = 26) included therapists who were most effective in terms of clients’ OQ-45 change scores and the corresponding effect sizes measured with Cohen’s *d*. The post-hoc comparisons revealed that both Cluster 2 and Cluster 4 were significantly different from Clusters 1 and 3 in terms of the OQ-45 difference scores. Furthermore, Cluster 4 had a significantly larger effect size *d* than Clusters 1 and 3; for Cluster 2, the difference was significant with Cluster 3 (but not 1). There was no significant difference between Clusters 1 and 3 or between Clusters 2 and 4 in terms of outcomes on the OQ-45 scores. It should be noted, however, that Cluster 4 had a high percentage of dropouts. In fact, the percentage of dropouts in Cluster 4 was significantly greater than in Cluster 2 or Cluster 3, albeit not significantly different from Cluster 1. At the same time, the number of sessions (i.e., treatment duration) for the therapists in Cluster 2 was significantly greater than for the therapists in Cluster 3 or Cluster 4.

Cluster 1, the smallest cluster (*n* = 9), included therapists with lower effectiveness (as measured with the OQ-45) but an average dropout rate. These therapists also had significantly more referrals than the therapists in Clusters 2, 3, or 4. The therapists in Cluster 3 (*n* = 17) were also less effective (as measured by the OQ-45), and they had average rates of dropouts and referrals. Nevertheless, these therapists stood out from the other therapists in that their clients expressed less satisfaction with the treatment than the clients of therapists in Cluster 4 or Cluster 2.

In summary, Cluster 4 included the largest number of therapists. They were the most effective in terms of the OQ-45 scores, and they had short treatment durations compared to the therapists in Cluster 2. However, given their high dropout rates, their effectiveness applied solely to the clients whom they retained. The therapists in Cluster 2, on the other hand, were the best therapists all-round; they were both highly effective and retained the most clients in treatment. Cluster 1 consisted of therapists who had average scores on most of the dimensions of outcome, but they had a notable tendency to refer clients elsewhere. Finally, the therapists in Cluster 3 were less effective than the therapists in the other clusters, and they had the lowest client satisfaction scores of all the therapists.

### Differences Among Therapists in the Different Clusters

We explored whether there were any differences in the general characteristics of the clusters in the entire group of therapists and the subset of 49 therapists who took part in Janse et al.‘s study (2023).We had access to more information about their characteristics and learning activities in the latter group.

There were no differences among the clusters in therapists’ gender or educational level. However, in Cluster 2 there were more master’s-level psychologists and therapists in training to become a certified psychologist (*N* = 7, 46.7%) than in the other clusters. These therapists, therefore, were less experienced than therapists in the other clusters. By contrast, Cluster 3 had the fewest therapists in training (*N* = 2, 11.8%).

The therapists in Clusters 2 and 4 had the fewest years of experience (*M* = 8.33, *SD* = 3.78 and *M* = 8.21, *SD* = 5.84, respectively), and the therapists in Cluster 3 had the most years of experience (*M* = 13.25, *SD* = 6 0.45), although the Kruskal-Wallis test indicated that the difference was not significant [H(3) = 5.89, *p* = .117]. In the subsample, the therapists indicated the amount of time they spend on learning activities. Therapists in Cluster 2 spent the most time per week, and those in Cluster 3 spent the least amount of time each week on learning activities (*M* = 5.89, *SD* = 6.55 versus *M* = 2.25, *SD* = 1.55), but the differences among the clusters was not significant [H (3) = 5.39, *p* = .146]. Cluster 2, however, spent significantly more time on their post-master’s education than the other clusters [H (3) = 15.13, *p* = .002], and pairwise comparisons using Dunn’s test showed that the largest difference was in Cluster 3 [19.74 (5.75), *p* = .004]. The clusters did not significantly differ in other learning activities.

## Discussion

This study aimed to unravel therapist effects (TEs) by identifying differences among therapists while controlling for various factors in a real-world treatment setting. Although studies on TEs often concentrate on clients’ symptom reduction as the treatment outcome, other measures are also clinically relevant for evaluating the quality of therapists’ performance. These measures include clients’ dropout rates, therapists’ referrals to other treatments, the duration of clients’ treatment, and clients’ satisfaction with treatment. Despite the study’s exploratory nature, we hypothesized that TEs would differ across various indicators of treatment outcome.

The results revealed that there were significant differences among therapists, even though this was a stable clinical environment in which clients were generally randomly assigned to therapists with similar professional training and a similar therapeutic orientation. The results from separate multilevel models supported this hypothesis. A descriptive comparison of the TEs indicated different TEs for different aspects of the treatment, as shown by the following percentages: Symptom reduction (2.6%), number of sessions (6.5%), clients’ dropout rates (4.7%), and therapists’ referrals to other treatments (5.1%). Even clients’ satisfaction scores indicated a stronger TE (3.8%) than the TE based on reduction in symptoms. Consequently, relying solely on symptom reduction as a gauge of TEs provides a limited perspective on therapists’ actual clinical performance. Correlations across the different measures of clients’ treatment outcome were often weak, and thus implied that a therapist who excelled in one area might not do so in other areas. This conclusion is consistent with previous research (Lutz et al., 2015; Barkham et al., 2017).

Although the impact of the different therapist effects may seem modest, they have significant methodological implications. Janse et al. (2023) made use of a sample from the same treatment center, but restricted their sample to therapists with at least 10 clients who had completed the OQ measures (at the start and end of treatment) and found TEs of 1.4%. This sample also had a smaller dropout and referral percentage than the current sample, which is not surprising as these clients less often had complete scores on the OQ-45. Our broader client inclusion criterion, i.e., including clients with missing data, led to an inclusion of more therapists and increased the magnitude of TEs. This result prompts these questions: Do the largest TEs become apparent only when extreme cases are included? Are clients who drop out of treatment prematurely those who benefit the most from working with the most skilled therapists? Answers to these questions call for further investigations.

By bringing together the different TEs into one cluster analysis, we found four types of therapists. The first cluster can be interpreted as *good for some.* Therapists in this cluster exhibited average performance in most areas. However, remarkably, they referred clients to other professionals or services. They might tend to abandon efforts in the presence of difficulties or, alternatively, prioritize clients’ overall well-being and seek optimal solutions, even if it involves referring them to other professionals or services. The second group could be described as *the talents*. This cluster comprised therapists who excelled in both effectiveness (as indicated by OQ-45 scores) and client retention and can be considered the most well-rounded practitioners. The third cluster can be described as *less effective with low satisfaction*: therapists in this cluster were less effective than their counterparts in other clusters. Furthermore, they had the lowest client satisfaction scores among all the therapists studied. Finally, the fourth cluster can be regarded as *efficient but high dropout rate*. This cluster included highly effective therapists in terms of OQ-45 scores. However, their impact was limited to the clients they retained due to a high dropout rate. Despite shorter treatment durations compared to the second cluster, their effectiveness was constrained by client retention challenges.

The differences among therapists described here could have significant implications for clients’ treatment. For specific types of clients, it might matter more who the therapist is, than for others. Some therapists seem to have a major positive impact on symptom reduction, whereas other therapists might be better equipped to retain clients in therapy. Only a small number of the therapists have both kinds of expertise. These findings should be considered in future studies aimed at personalizing treatment for clients.

Despite their significance, we lack solid evidence regarding the therapist characteristics that drive these differences. Neither therapists’ age nor gender appears to be decisive. While dedicating time to post-master education, learning activities, and experience might play a role, the tendency for less experienced psychologists in training (Clusters 2 and 4) to engage in more learning activities complicates the interpretation of the relationship between therapists’ experience and their clients’ outcomes. Thus, the strength of these factors’ contribution to Treatment Effects (TEs) is inconclusive. Other potential factors include therapists’ interpersonal skills (Anderson et al., 2016) and their responsiveness (Stiles & Horvath, 2017). Additionally, TEs may be shaped by the interplay between client and therapist attributes, particularly in terms of how well they are matched. Further research will be required to untangle these possibilities.

This study was the first to demonstrate the multifaceted relationships among therapist characteristics, clients’ treatment outcomes, and therapists’ performance; nevertheless, limitations do exist. The primary limitation was the relatively small number of therapists who participated. Although 68 therapists, combined with a large number of clients, was sufficient to investigate therapists’ effects on clients’ treatment outcome, this number was modest for examining differences among the therapist subgroups. Expanding the pool of therapists and replicating the clusters that were identified through latent profile analysis would be a logical next step. Another limitation relates to the incomplete OQ-45 scores for clients who discontinued treatment or were referred elsewhere. For instance, although Cluster 4 showed effectiveness in case-mix-corrected OQ-45 change scores, high dropout rates were observed. Cluster 1 had a high referral rate. Complete OQ-45 change scores might have revealed reduced effectiveness for Clusters 4 and 1, potentially altering cluster formation. As a potential solution, we considered multilevel imputation; however, this approach should be used with caution due to the likelihood of cross-level interactions and non-linearity. Another limitation is that the reasons for prematurely discontinuing treatment and referrals were unknown. While valid reasons for referring patients to alternative treatments may exist, therapists exhibit varying approaches in persisting with challenging cases. Some may be committed to treating patients despite difficulties, while others may choose to discontinue efforts earlier, perhaps recognizing the potential benefits of alternative treatment modalities.

Further research is necessary on the reasons for termination and TEs. However, it is interesting to see differences between therapists in this regard within the same facility. Despite these limitations, this study generated important hypotheses that warrant being tested in future research.

In sum, this exploratory study aimed to delve deeper into therapist effects and how therapists’ performances are viewed. The study provided evidence that all therapists are not equally effective and the influence of therapists extends beyond mere symptom reduction or improvements in general functioning. This is important for further consideration in future investigations of therapist effects and the characteristics of effective therapists.

## Electronic Supplementary Material

Below is the link to the electronic supplementary material.

Supplementary Material 1
