# Supplementary material for: Disentangling the Therapist Effect: Clustering Therapists by Using Different Treatment Outcomes
Source: Adm Policy Ment Health. 2024 Mar 21;51(5):769–79. doi: 10.1007/s10488-024-01365-3 (PMC11379780; doi:10.1007/s10488-024-01365-3)
Supplement: Supplementary file 1 — Supplementary Material 1 [file 10488_2024_1365_MOESM1_ESM.docx]

**ONLINE SUPPLEMENTAL MATERIALS**

**Results of the multi-level analyses of the different treatment outcomes**

Table 1.a Multi-level model with OQ change scores as outcome with no predictors

| Parameter | Estimate | SE | *t* | *p* | 95% | CI |
| --- | --- | --- | --- | --- | --- | --- |
| *Fixed effects* |  |  |  |  |  |  |
| Intercept | 19.10 | .65 | 29.39 | <.001 | 17.80 | 20.40 |
| *Covariance parameters* |  |  | *z* |  |  |  |
| Residual | 469.87 | 13.54 | 34.69 | <.001 | 444.06 | 497.17 |
| Intercept variance* | 12.31 | 4.70 | 2.62 | .019 | 3.83 | 26.03 |
| ICC = .026 -2 Log Likelihood= 22238.52 AIC = 22244.52 BIC = 22261.95 | | | | | | |

*subject = therapist

Table 1.b. Multi-level model with OQ change scores as outcome with covariates

| Parameter | Estimate | SE | *t* | *p* | 95% | CI |
| --- | --- | --- | --- | --- | --- | --- |
| *Fixed effects* |  |  |  |  |  |  |
| Intercept | 16.54 | .82 | 20.22 | <.001 | 14.93 | 18.15 |
| OQ pre-treatment | .30 | .02 | 15.63 | <.001 | .27 | .34 |
| Clients Age | -.08 | .03 | -2.74 | .006 | -.14 | -.02 |
| Symptom duration | -1.75 | .28 | -6.27 | <.001 | -2.29 | -1.20 |
| Employment | 4.36 | .89 | 4.91 | <.001 | 2.62 | 6.10 |
| *Covariance parameters* |  |  | *z* |  |  |  |
| Residual | 418.80 | 12.12 | 34.56 | <.001 | 395.71 | 443.24 |
| Intercept variance* | 8.75 | 3.82 | 2.29 | .022 | 3.72 | 20.58 |
| ICC = .021 -2 Log Likelihood= 21788.25 AIC = 21802.25 BIC = 21842.87 | | | | | | |

* subject = therapist

Table 1.c. Multi-level model with OQ change scores as outcome with covariates and random slope

| Parameter | Estimate | SE | | *t* | *p* | | 95% | | CI |
| --- | --- | --- | --- | --- | --- | --- | --- | --- | --- |
| *Fixed effects* |  |  | |  |  | |  | |  |
| Intercept | 16.83 | .82 | | 20.56 | <.001 | | 15.22 | | 18.43 |
| OQ pre-treatment | .32 | .03 | | 11.97 | <.001 | | .27 | | .37 |
| Clients Age | -.08 | .03 | | -2.84 | .004 | | -.14 | | -.03 |
| Symptom duration | -1.72 | .28 | | -6.20 | <.001 | | -2.26 | | -1.17 |
| Employment | 4.40 | .88 | | 4.98 | <.001 | | 2.67 | | 6.13 |
| *Covariance parameters* |  |  | | *z* |  | |  | |  |
| Residual | 413.53 | 12.27 | | 33.71 | <.001 | | 390.17 | | 438.28 |
| Intercept variance | 9.04 | 4.02 | | 2.25 | .024 | | 3.79 | | 21.59 |
| Covariance intercept and slope | .36 | .14 | 2.57 | | .010 | .09 | | .63 | |
| Variance of the slope | .00 | .01 | .04 | | .964 | .00 | | .00 | |
| -2 Log Likelihood= 21770.68 AIC = 21788.68 BIC = 21840.92 | | | | | | | | | |

a. This covariance parameter is redundant. The test statistic and confidence interval cannot be computed.

Table 2.a. Multi-level model with sessions as outcome with no predictors

| Parameter | Estimate | SE | *t* | *p* | 95% | CI |
| --- | --- | --- | --- | --- | --- | --- |
| *Fixed effects* |  |  |  |  |  |  |
| Intercept | 8.31 | .14 | 60.28 | <.001 | 8.03 | 8.58 |
| *Covariance parameters* |  |  | *z* |  |  |  |
| Residual | 13.48 | .26 | 52.49 | <.001 | 12.98 | 13.99 |
| Intercept variance* | 1.04 | .23 | 4.54 | <.001 | .67 | 1.59 |
| ICC = .071 -2 Log Likelihood= 30479.10 AIC = 30485.10 BIC = 30504.98 | | | | | | |

*subject = therapist

Table 2.b. Multi-level model with sessions as outcome with no predictors (excluding clients who had dropped out of treatment).

| Parameter | Estimate | SE | *t* | *p* | 95% | CI |
| --- | --- | --- | --- | --- | --- | --- |
| *Fixed effects* |  |  |  |  |  |  |
| Intercept | 8.84 | .13 | 68.18 | <.001 | 8.58 | 9.10 |
| *Covariance parameters* |  |  | *z* |  |  |  |
| Residual | 12.44 | .26 | 47.73 | <.001 | 11.93 | 12.96 |
| Intercept variance* | .87 | .20 | 4.29 | <.001 | .55 | 1.37 |
| ICC = .065 -2 Log Likelihood= 24910.54 AIC = 24916.54 BIC = 24935.86 | | | | | | |

*subject = therapist

Table 2.c. Multi-level model with sessions as outcome with covariates (excluding clients who had dropped out of treatment).

| Parameter | Estimate | SE | *t* | *p* | 95% | CI |
| --- | --- | --- | --- | --- | --- | --- |
| *Fixed effects* |  |  |  |  |  |  |
| Intercept | 8.73 | .14 | 60.38 | <.001 | 8.44 | 9.02 |
| OQ pre-treatment | .02 | .00 | 8.59 | <.001 | .02 | .03 |
| Client’s age | -.01 | .00 | -1.88 | .060 | -.02 | .00 |
| Symptom duration | .04 | .04 | 1.03 | .301 | -.04 | .11 |
| Employment | .16 | .12 | 1.34 | .179 | -.07 | .40 |
| *Covariance parameters* |  |  | *z* |  |  |  |
| Residual | 12.28 | .28 | 43.39 | <.001 | 11.73 | 12.84 |
| Intercept variance* | .72 | .18 | 3.99 | <.001 | .44 | 1.17 |
| ICC = .055 -2 Log Likelihood= 20595.04 AIC = 20609.04 BIC = 20652.81 | | | | | | |

*subject = therapist

Table 3.a. Multi-level model with client satisfaction scores as outcome

| Parameter | Estimate | SE | *t* | *p* | 95% | CI |
| --- | --- | --- | --- | --- | --- | --- |
| *Fixed effects* |  |  |  |  |  |  |
| Intercept | 8.20 | .05 | 171.33 | <.001 | 8.10 | 8.29 |
| *Covariance parameters* |  |  | *z* |  |  |  |
| Residual | 2.29 | .06 | 39.37 | <.001 | 2.18 | 2.40 |
| Intercept variance* | .09 | .03 | 3.17 | .002 | .05 | .16 |
| ICC = .038 -2 Log Likelihood= 11681.21 AIC = 11687.21 BIC = 11705.39 | | | | | | |

*subject = therapist

Table 3.b. Multi-level model with client satisfaction scores as outcome with covariates

| Parameter | Estimate | SE | *t* | *p* | 95% | CI |
| --- | --- | --- | --- | --- | --- | --- |
| *Fixed effects* |  |  |  |  |  |  |
| Intercept | 8.10 | .06 | 133.04 | <.001 | 7.98 | 8.22 |
| OQ pre-treatment | -.01 | .00 | -6.17 | <.001 | -.01 | -.01 |
| Client’s age | .00 | .00 | 2.10 | .036 | .00 | .01 |
| Symptom duration | -.08 | .02 | -3.97 | <.001 | -.11 | -.04 |
| Employment | .15 | .06 | 2.43 | .015 | .03 | .27 |
| *Covariance parameters* |  |  | *z* |  |  |  |
| Residual | 2.15 | .06 | 35.82 | <.001 | 2.03 | 2.27 |
| Intercept variance* | .07 | .02 | 2.85 | .004 | .03 | .13 |
| ICC = .032 2 Log Likelihood= 9519.95 AIC = 9533.95 BIC = 9575.07 | | | | | | |

*subject = therapist

Table 4.a. Multi-level model with drop out

| Parameter | Estimate | SE | *t* | *p* | 95% | CI |
| --- | --- | --- | --- | --- | --- | --- |
| *Fixed effects* |  |  |  |  |  |  |
| Intercept | -1,78 | ,07 | -26.90 | <,001 | -1,92 | -1,65 |
| *Random effect covariance* |  |  | *z* |  |  |  |
| Intercept variance | .16 | .06 | 2.83 | .005 | .08 | .33 |
| AIC = 26722.05 BIC = 26728.66 | | | | | | |

Table 4.b. Multi-level model with drop out as outcome with covariates

| Parameter | Estimate | SE | *t* | *p* | 95% | CI |
| --- | --- | --- | --- | --- | --- | --- |
| *Fixed effects* |  |  |  |  |  |  |
| Intercept | -1.52 | .09 | -16.64 | <.001 | -1.70 | -1.34 |
| OQ pre-treatment | .00 | .00 | .93 | .352 | .00 | .01 |
| Client’s age | -.01 | .00 | -5.33 | <.001 | -.02 | -.01 |
| Symptom duration | .09 | .03 | 3.43 | <.001 | .04 | .15 |
| Employment | -.46 | .10 | -4.63 | <.001 | -.66 | -.27 |
| *Random effect covariance* |  |  | *z* |  |  |  |
| Intercept variance | .18 | .07 | 2.80 | .005 | .09 | .37 |
| AIC = 22371.45 BIC = 22377.88 | | | | | | |

Table 5.a. Multi-level model with referrals as outcome

| Parameter | Estimate | SE | *t* | *p* | 95% | CI |
| --- | --- | --- | --- | --- | --- | --- |
| *Fixed effects* |  |  |  |  |  |  |
| Intercept | -2.05 | .07 | -30.00 | <.001 | -2.19 | -1.92 |
| *Random effect covariance* |  |  | *z* |  |  |  |
| Intercept variance | .16 | .05 | 3.12 | .002 | .08 | .30 |
| AIC = 28149.79 BIC = 28156.39 | | | | | | |

Table 5.b. Multi-level model with referrals as outcome with covariates

| Parameter | Estimate | SE | *t* | *p* | 95% | CI |
| --- | --- | --- | --- | --- | --- | --- |
| *Fixed effects* |  |  |  |  |  |  |
| Intercept | -1.93 | .10 | -19.76 | <.001 | -2.12 | -1.74 |
| OQ pre-treatment | .03 | .00 | 10.60 | <.001 | .02 | .03 |
| Client’s age | .00 | .00 | -1.57 | .12 | -.01 | .00 |
| Symptom duration | .06 | .03 | 1.67 | .09 | -.01 | .12 |
| Employment | -.43 | .11 | -3.98 | <.001 | -.64 | -.22 |
| *Random effect covariance* |  |  | *z* |  |  |  |
| Intercept variance | .16 | .06 | 2.75 | .006 | .08 | .32 |
| AIC = 24201.90 BIC = 24208.33 | | | | | | |

**Results of the calculation of optimal number of clusters**


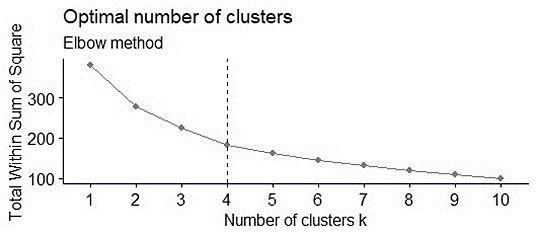


Figure 1. Optimal number of clusters based on the Elbow method


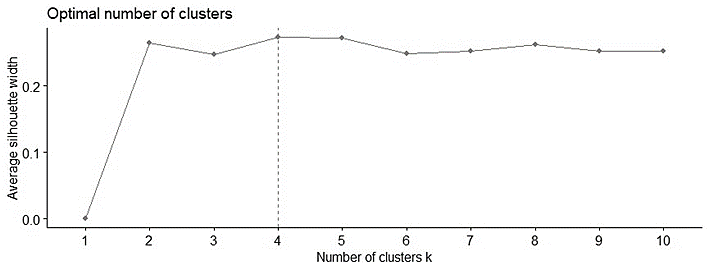


Figure 2. Optimal number of clusters based on the silhouette method

**Results of the post-hoc tests comparing the clusters on outcomes**

Table 6. Pairwise comparisons of the clusters on the OQ-45 change scores

| Cluster comaprison | Test Statistic | SE | Std. Test Statistic | Adj. Sig.^a^ |
| --- | --- | --- | --- | --- |
| 4-2 | .39 | 6.32 | .06 | 1.000 |
| 4-1 | 25.13 | 7.54 | 3.33 | .006 |
| 4-3 | 31.52 | 6.08 | 5.19 | <.001 |
| 2-1 | 24.73 | 8.22 | 3.01 | .016 |
| 2-3 | -31.13 | 6.90 | -4.51 | <.001 |
| 1-3 | -6.39 | 8.03 | -.80 | 1.000 |

^a^ Significance values have been adjusted by the Bonferroni correction for multiple tests.

Table 7. Pairwise comparisons of the clusters on the effect size *d*

| Cluster comaprison | Test Statistic | SE | Std. Test Statistic | Adj. Sig. |
| --- | --- | --- | --- | --- |
| 3-1 | 7.44 | 8.03 | .926 | 1.000 |
| 3-4 | -27.38 | 6.08 | -4.51 | <.001 |
| 3-2 | 27.95 | 6.90 | 4.05 | <.001 |
| 1-4 | -19.94 | 7.54 | -2.65 | .049 |
| 1-2 | -20.51 | 8.23 | -2.50 | .075 |
| 4-2 | .57 | 6.32 | .09 | 1.000 |

Table 8. Pairwise comparisons of the clusters on the dropout percentages

| Cluster comaprison | Test Statistic | SE | Std. Test Statistic | Adj. Sig. |
| --- | --- | --- | --- | --- |
| 2-3 | -17.49 | 6.90 | -2.53 | .068 |
| 2-1 | 21.52 | 8.22 | 2.62 | .053 |
| 2-4 | -39.44 | 6.32 | -6.24 | <.001 |
| 3-1 | 4.04 | 8.03 | .50 | 1.000 |
| 3-4 | -21.96 | 6.08 | -3.61 | .002 |
| 1-4 | -17.92 | 7.54 | -2.38 | .104 |

Table 9. Pairwise comparisons of the clusters on the referral percentages

| Cluster comaprison | Test Statistic | SE | Std. Test Statistic | Adj. Sig. |
| --- | --- | --- | --- | --- |
| 3-4 | -6.19 | 6.08 | -1.02 | 1.000 |
| 3-2 | 6.73 | 6.90 | .98 | 1.000 |
| 3-1 | 37.89 | 8.03 | 4.72 | .000 |
| 4-2 | .54 | 6.32 | .09 | 1.000 |
| 4-1 | 31.70 | 7.54 | 4.21 | .000 |
| 2-1 | 31.16 | 8.22 | 3.79 | .001 |

Table 10. Pairwise comparisons of the clusters on client satisfaction ratings

| Cluster comaprison | Test Statistic | Std. Error | Std. Test Statistic | Adj. Sig. |
| --- | --- | --- | --- | --- |
| 3-4 | -16.95 | 6.08 | -2.79 | .032 |
| 3-1 | 16.97 | 8.03 | 2.11 | .208 |
| 3-2 | 18.11 | 6.90 | 2.62 | .052 |
| 4-1 | .02 | 7.54 | .00 | 1.000 |
| 4-2 | 1.16 | 6.32 | .18 | 1.000 |
| 1-2 | -1.144 | 8.22 | -.14 | 1.000 |

Table 11. Pairwise comparisons of the clusters on number of sessions

| Cluster comaprison | Test Statistic | SE | Std. Test Statistic | Adj. Sig. |
| --- | --- | --- | --- | --- |
| 4-3 | 1.13 | 6.08 | .19 | 1.000 |
| 4-1 | 14.71 | 7.54 | 1.95 | .306 |
| 4-2 | 19.36 | 6.31 | 3.07 | .013 |
| 3-1 | 13.58 | 8.03 | 1.69 | .545 |
| 3-2 | 18.24 | 6.92 | 2.64 | .049 |
| 1-2 | -4.66 | 8.22 | -.57 | 1.000 |
